# The Role of Leptin in Childhood Immune Thrombocytopenia (ITP): An Anti-Inflammatory Agent?

**DOI:** 10.3390/ijms22147636

**Published:** 2021-07-16

**Authors:** Iason Thomas, Ioannis Panagoulias, Ioanna Aggeletopoulou, Anastasia Varvarigou, Bessie E. Spiliotis, Athanasia Mouzaki

**Affiliations:** 1 Laboratory of Immunohematology, Division of Hematology, Department of Internal Medicine, Medical School, University of Patras, GR-26500 Patras, Greece; iason.thomas@doctors.org.uk (I.T.); iopanagoulia@upatras.gr (I.P.); iaggel@hotmail.com (I.A.); 2 Allergy Centre, Wythenshawe Hospital, Manchester University NHS Foundation Trust, Manchester M23 9LT, UK; 3 Department of Paediatrics, Medical School, Patras University Hospital, University of Patras, GR-26500 Patras, Greece; varvarigou@upatras.gr (A.V.); besspil@endo.gr (B.E.S.)

**Keywords:** leptin, childhood immune thrombocytopenia (ITP), peripheral blood mononuclear cells, T cells, monocytes, cytokines, gene expression

## Abstract

To investigate the effect of leptin in childhood ITP, we measured plasma leptin in 39 children with acute ITP, after treatment and in remission, and in 33 healthy age/BMI-matched controls. We also cultured ITP and control peripheral blood mononuclear cells (PBMCs) with recombinant leptin to assess its direct effect on pro/anti-inflammatory cytokine gene expression. A significant increase in leptin was observed in children with active disease compared to controls. A significant inverse correlation of leptin with platelet count was also observed in children with acute ITP. Leptin remained high after treatment with IVIg, whereas steroid treatment lowered leptin below control levels. In remission, leptin was in the control range. Cytokine gene expression was significantly increased in children with acute ITP compared with controls, with highest expression for IFN-γ and IL-10. IVIg/steroid treatment significantly decreased IFN-γ and IL-10 expression. In remission, IFN-γ and IL-10 expression remained low. Addition of leptin to PBMCs isolated from patients in remission resulted in a significant increase in IL-10 gene expression compared to controls. Further experiments with purified T-cells and monocytes identified monocytes as the source of leptin-induced IL-10. We suggest that leptin acts as an active anti-inflammatory agent in childhood ITP by promoting IL-10 secretion by monocytes.

## 1. Introduction

Leptin is a hormone encoded by the Ob gene [1] and produced mainly, but not exclusively, by adipocytes. It is a non-glycosylated 16 kD protein consisting of 146 amino acids. The leptin receptor (OB-R or LEP-R) shows sequence homology and functional similarity to the class I cytokine receptor family and is expressed in six different isoforms [2,3]. Leptin plays a role in metabolism, often paired with the hormone ghrelin, and regulates energy balance by signaling satiety in the hypothalamus [4]. Plasma levels of leptin are closely related to body mass index (BMI) and fat mass [5]. However, leptin has been shown to be more than a satiety signal or energy indicator. Leptin receptors are found primarily in the central nervous system, but also in a variety of other cell types in the periphery, such as cells of the reproductive, hematopoietic, and immune systems [6,7,8,9,10].

Leptin has been shown to influence innate and adaptive immunity [6,10,11]. It acts as a chemoattractant for neutrophils, eosinophils, and basophils [12,13,14]. Leptin signaling induces phagocytosis [15] and also promotes the production of pro- and anti-inflammatory cytokines by monocytes and macrophages [15,16]. Under certain conditions, leptin can stimulate basophils to secrete the type 2 cytokines IL-4 and IL-13 [12]. Leptin also influences dendritic cell maturation [17], regulates mast cell cytokine production [18], and may have an inhibitory effect on natural killer (NK) cell activation [19]. Leptin also appears to play a role in T cell differentiation. In particular, leptin receptor signaling in T helper cells is required for Th17 differentiation [20] but may also suppress regulatory T cell (Treg) differentiation [21].

Although adipocytes are the main cells that produce leptin, Tregs and effector T helper cells have been shown to produce leptin upon activation [22,23]. As such, immune cell-derived leptin could act as an autocrine and/or paracrine signal that can enhance the action of leptin through the leptin receptor expressed on immune cells. Elevated levels of circulating leptin have also been associated with acute infections [24] and certain autoimmune diseases, including immune thrombocytopenia (ITP) [25,26,27,28]. It has previously been suggested that leptin plays an important role in the pathogenesis of ITP, and it has been proposed as a potential treatment target [26,27,28].

ITP is an autoimmune disease that affects children and adults. It is characterized by isolated thrombocytopenia (peripheral platelet count < 100 × 10^9^/L) in the absence of other causes [29]. ITP is mediated by platelet-specific autoantibodies that facilitate phagocytosis of opsonized platelets by Fc-γ receptor (FcγR)-carrying phagocytes, predominantly in the spleen [30]. In adults, ITP is usually chronic, whereas in children the disease is usually acute. The majority of children present with acute development of purpura and bruising, often following viral infection. Approximately two-thirds of children recover within 6 months of onset. The rate of persistent or chronic disease, defined by a platelet count < 150 × 10^9^/L at 6 months, appears to increase with age [29]. In children with chronic ITP, the prevalence of severe thrombocytopenia (peripheral platelet count < 20 × 10^9^/L) is low. According to current recommendations [31], the initial management of newly diagnosed childhood ITP, regardless of platelet count, consists of careful observation when there is little or no bleeding (skin manifestations). Drug therapy with corticosteroids, intravenous immunoglobulin (IVIg) or anti-D immunoglobulin, either alone or in combination, is usually reserved for children with more severe bleeding. Thrombopoietin receptor agonists, rituximab and/or splenectomy may be considered as second-line therapies for non-responders [31].

Patients with chronic ITP show increased Th1 cells both in the circulation and in the spleen, which mainly secrete IFN-γ [32,33,34]. Th1 polarization is important for macrophage stimulation and probably promotes platelet phagocytosis. Other T helper cell subsets, such as Th17 and Th22, have also been implicated in the pathogenesis of ITP [30], as has regulatory T cell (Treg) dysfunction [35,36].

Most children with newly diagnosed ITP express the cytokines IL-2 and IFN-γ (with or without IL-4) in vivo, indicating a Th0 pattern or early Th1 cell activation [37]. Stable remission is associated with a Th0 or Th2 pattern, whereas persistent expression of IFN-γ indicates the development of a cyclic or chronic form of ITP. Important distinguishing factors between the milder relapsing form and the aggressive chronic form of ITP are the relative intensity of IFN-γ expression and the presence or absence of the anti-inflammatory cytokine IL-10 [34,37]. IL-10 provides a negative feedback mechanism that inhibits Th1 cell and monocyte/macrophage activation [38]; therefore, the complete absence of IL-10 expressing cells in peripheral blood likely contributes to increased Th1 cytokine synthesis by autoreactive T cell clones.

In this work, we investigated the role of leptin in childhood ITP, an autoimmune disease that has clear stages that can be followed closely by platelet counts, and no underlying pathology. To this end, we measured plasma leptin in children with acute ITP, after treatment and in remission, and in healthy controls. We also cultured ITP and control peripheral blood mononuclear cells (PBMCs) with recombinant leptin to assess its direct effect on pro- and anti-inflammatory cytokine gene expression.

## 2. Results

### 2.1. Plasma Leptin and TGF-β Levels

The data of the study participants are presented in Table 1.

**Figure 1:**
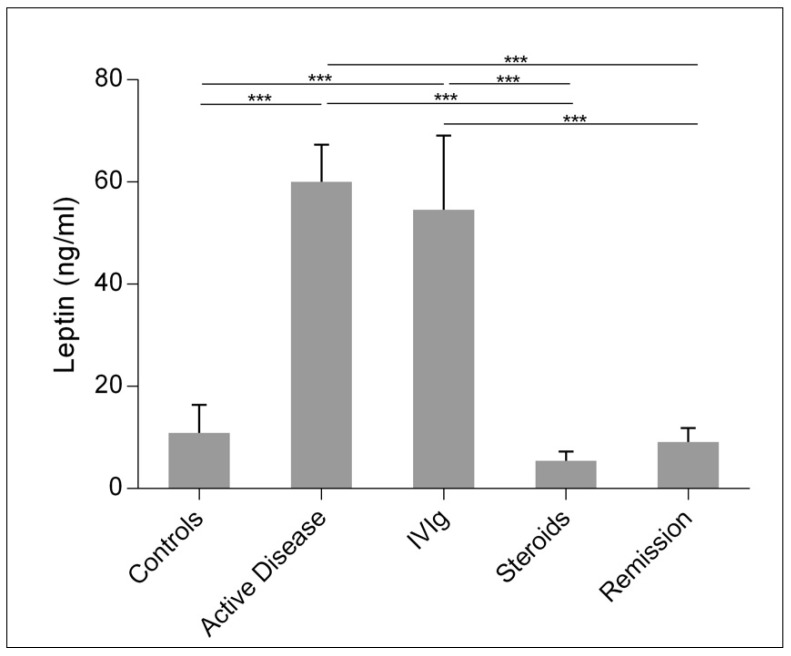
Plasma leptin levels in controls and children with ITP in the acute phase, after treatment with IVIg and/or steroids, and in remission. Leptin levels are shown as mean (SD). Asterisks indicate statistical significance (*** *p* < 0.001).

Measurement of plasma leptin levels in patients and controls (Figure 1) revealed a significant increase in leptin levels in children with acute ITP compared with controls. Treatment with intravenous immunoglobulin (IVIg) resulted in a slight, nonsignificant decrease in leptin levels, whereas steroid treatment as add-on or monotherapy resulted in a statistically significant decrease in leptin levels below control levels. In remission, leptin levels were in the control range.

**Figure 2:**
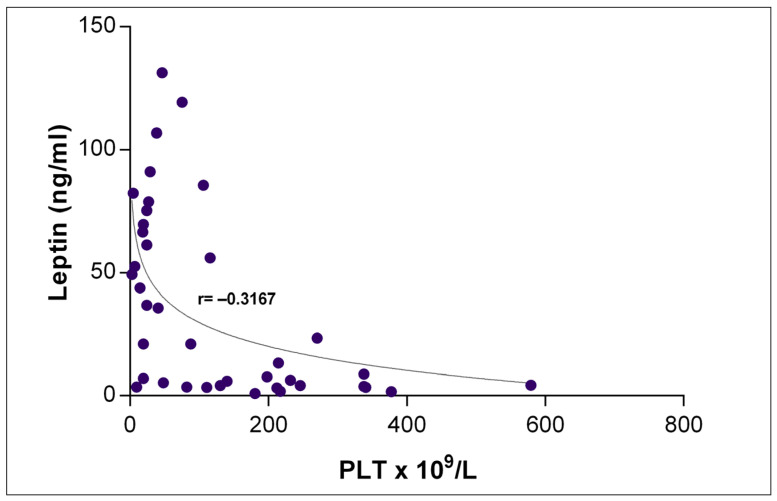
Correlation of plasma leptin levels and platelet (PLT) count.

We also found a significant inverse correlation of leptin levels with peripheral platelet count in children with acute ITP (Figure 2).

**Figure 3:**
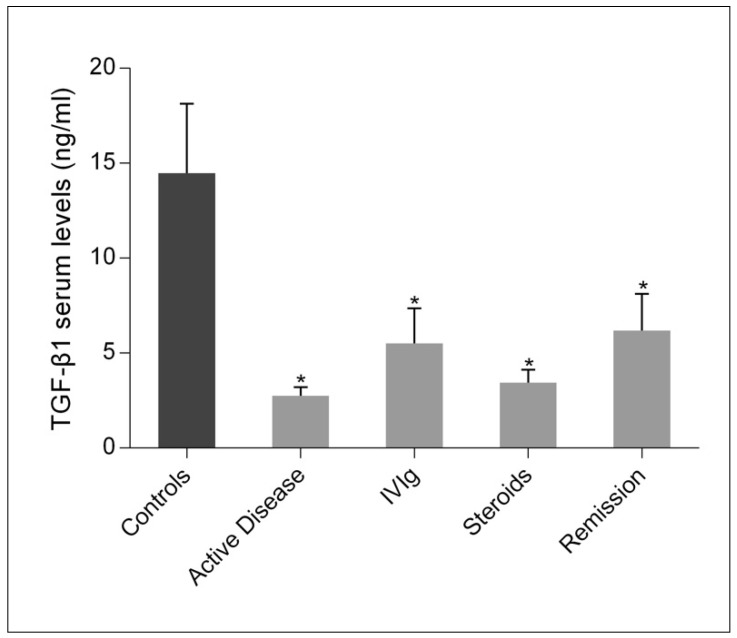
Plasma TGF-β1 levels in controls and children with ITP in the acute phase, after treatment with IVIg and/or steroids, and in remission. TGF-β1 levels are shown as mean (SD). Asterisks indicate statistically significant differences (* *p* < 0.05) compared to controls.

Measurement of plasma TGF-β1 levels in patients and controls (Figure 3) revealed that TGF-β1 levels were significantly lower in children with acute ITP compared with controls and remained significantly lower in patients on IVIg and/or steroid therapy or even when the disease went into remission.

### 2.2. Ex-Vivo Cytokine Gene Expression

**Figure 4:**
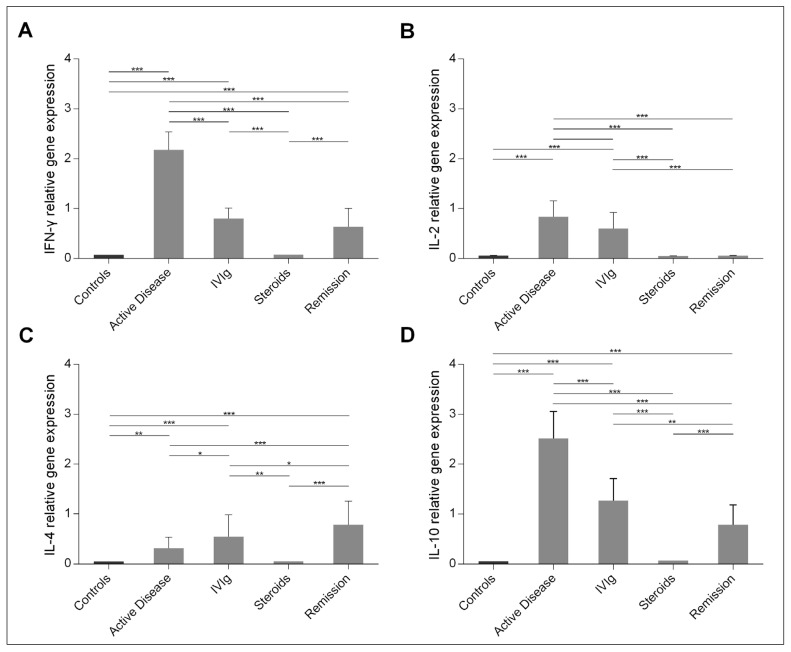
Ex-vivo expression of (**A**) IFN-γ, (**B**) IL-2, (**C**) IL-4 and (**D**) IL-10 in PBMCs isolated from controls and children with ITP in the acute phase, after treatment with IVIg and/or steroids, and in remission. Cytokine gene expression is shown as mean (SD). Asterisks indicate statistical significance (* *p* < 0.05; ** *p* < 0.01; *** *p* < 0.001).

The expression of cytokines IL-2, IFN-γ, IL-4 and IL-10 was studied in peripheral blood mononuclear cells (PBMCs) isolated from whole blood and processed immediately. The results (Figure 4) show that the expression of IL-2, IFN-γ, IL-4 and IL-10 was significantly increased in children with acute ITP compared to controls. The highest expression was observed for IFN-γ and IL-10 in the acute phase. Treatment with IVIg resulted in a significant decrease in IFN-γ and IL-10 expression and a significant increase in IL-4 expression, whereas treatment with steroids reduced the level of gene expression for all cytokines to control values. In remission, IFN-γ and IL-10 expression increased again but remained low; IL-4 expression reached the highest relative levels, while IL-2 expression was at control levels.

### 2.3. Effect of Leptin on Cytokine Gene Expression

**Figure 5:**
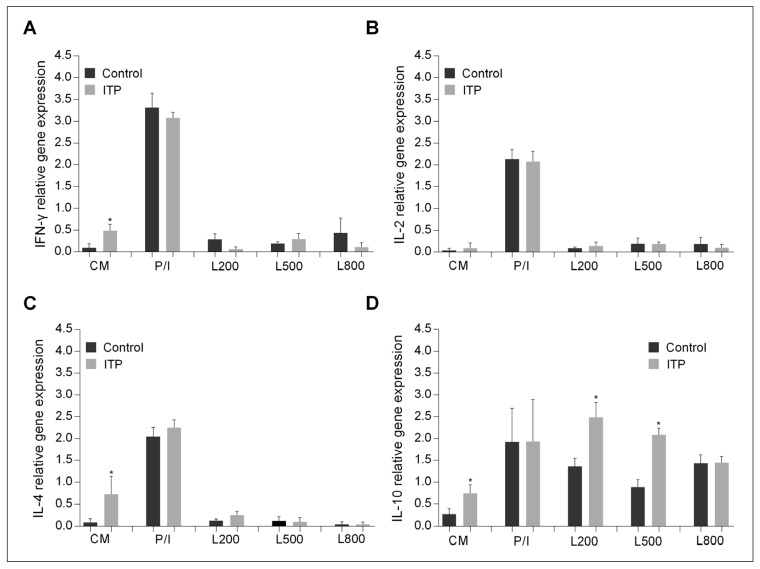
Effect of leptin on the expression of (**A**) IFN-γ, (**B**) IL-2, (**C**) IL-4 and (**D**) IL-10 in PBMCs isolated from controls and children with ITP in remission and cultured in plain culture medium (CM) or in the presence of phorbol myristate acetate and ionomycin (PI) or in the presence of recombinant human leptin at a concentration of 200 (L200) or 500 (L500) or 800 (L800) ng/mL. Cytokine gene expression is shown as mean (SD). Asterisks indicate statistically significant differences (* *p* < 0.05) compared to controls under the same culture conditions.

PBMCs isolated from ITP patients in remission and controls were cultured for 12 h in the presence or absence of the mitogens phorbol myristate acetate and ionomycin or recombinant human leptin at a concentration of 200 or 500 or 800 ng/mL (see M&M Section 4). At the end of the culture, the expression of the cytokines IL-2, IFN-γ, IL-4 and IL-10 was determined. The results (Figure 5) show that mitogenic stimulation of cells increased the expression of all cytokines in PBMCs from both patients and controls equally. Culture with leptin resulted in an increase in IL-10 gene expression in PBMCs from patients and controls. Comparison of the level of IL-10 gene expression between patients and controls showed that it was significantly higher in PBMCs from patients cultured with 200 or 500 ng/mL leptin.

### 2.4. Determination of the Cellular Source of Leptin-Induced IL-10

**Figure 6:**
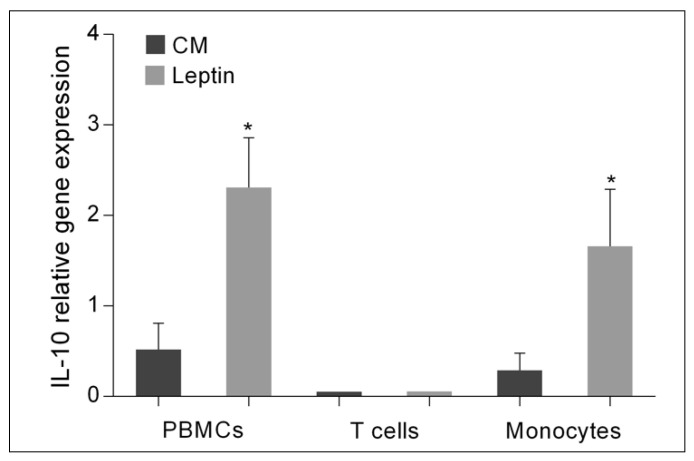
Effect of leptin on IL-10 gene expression in T cells and monocytes isolated from PBMCs from controls and ITP patients in remission that were cultured in plain culture medium (CM) or in the presence 200 ng/mL recombinant human leptin. IL-10 gene expression is shown as mean (SD). Asterisks indicate statistically significant differences (* *p* < 0.05) compared to controls.

To determine which PBMCs highly express IL-10 when cultured with leptin, we cultured PBMCs isolated from ITP patients in remission with 200 ng/mL of leptin for 12 h. At the end of culture, CD3+ T cells and CD14+ monocytes were isolated and analyzed for IL-10 gene expression. The results (Figure 6) show that leptin induces IL-10 expression only in monocytes.

## 3. Discussion

In humans, leptin has been associated with autoimmunity [39,40] mainly because elevated leptin levels have been observed in rheumatoid arthritis, systemic lupus erythematosus, psoriasis, inflammatory bowel disease, and ITP [25,26,27,28]. In infections, leptin has been associated with type 1 (pro-inflammatory) cytokine polarization [24,41], although under certain conditions it has been shown to enhance type 2 (anti-inflammatory) cytokine activity [12,16].

Childhood ITP does not appear to fully meet the definition of an established autoimmune disease with a polarized type 1 or type 2 response. As previously reported [37,42,43,44], acute childhood ITP presents with a Th0 or Th1 pattern and, in most cases, low levels of IL-4 and IL-6 that lead to increased expression of HLA-DR molecules on platelet surfaces. Sustained high expression of IFN-γ and resistance to IVIg treatment have been associated with poor prognosis, whereas a Th2 pattern and IL-10 expression are indicators of stable remission.

In agreement with previous studies [26,27,28,45], our results showed an increase in circulating leptin levels in children with acute ITP compared to healthy controls. Leptin levels appear to be negatively correlated with platelet count, as there is a positive correlation of plasma leptin with platelet-associated antibodies PAIgG and PAIgM in the acute phase.

Our results also showed that plasma TGF-β1 levels were decreased in all phases of the disease, in agreement with a previous report [46]. This suppression could be due to the reduced number and function of Treg cells that secrete TGF-β1 (Th3 cells) [47], but also to thrombocytopenia itself, since TGF-β1 from platelets contributes to plasma levels of TGF-β1 [48].

Consistent with previous studies [34,37,43,49], levels of IL-10 and IFN-γ were elevated, whereas IL-4 gene expression was low in newly diagnosed childhood ITP. IL-10 expression was reported to be significantly higher in children with acutely resolving ITP than in those whose disease progressed to chronic. IL-10 levels have been suggested to be a predictive biomarker of disease progression [37,42].

IL-10 is a cytokine produced by various cells, including monocytes/macrophages, dendritic cells, B cells, T helper and regulatory cells, and NK cells [50]. Its main function is to limit the inflammatory response. IL-10 inhibits the production of pro-inflammatory cytokines by Th1 cells via inhibition of activated macrophages [51,52] and can also suppress the antigen presentation ability of dendritic cells, monocytes and macrophages [53] and inactivate effector T helper cells via both self-regulation and regulation by Tregs [54]. In addition, IL-10 is secreted by type I Tregs (Tr1 cells) in humans [55]. Tr1 cells suppress antigen-specific effector T cell responses via IL-10 secretion and induce immune tolerance [56]. Therefore, high levels of IL-10 in children with ITP could protect them from an exaggerated inflammatory response and lead them into stable remission. Increased IL-10 gene expression in remission, together with increased IL-4, may counterbalance the pro-inflammatory IFN-γ effect, especially since TGF-β1 levels remain partially suppressed [38,43].

As shown in this work, leptin increases IL-10 gene expression by monocytes but not by T-cells in childhood ITP, as demonstrated from experiments with purified cells. For the experiments that examined the direct effect of leptin on PBMCs, T cells, and monocytes, we used blood samples from ITP patients in remission to assess the effect of leptin on cytokine gene expression in cells that were not overly activated by an ongoing acute immune response or their function altered or inhibited by drugs. We also focused on studying the expression of the cytokine genes IL-2, IFN-γ, IL-4, and IL-10 because previous studies have highlighted the importance of their expression patterns in disease progression [34,37,42,43,49].

**Figure 7:**
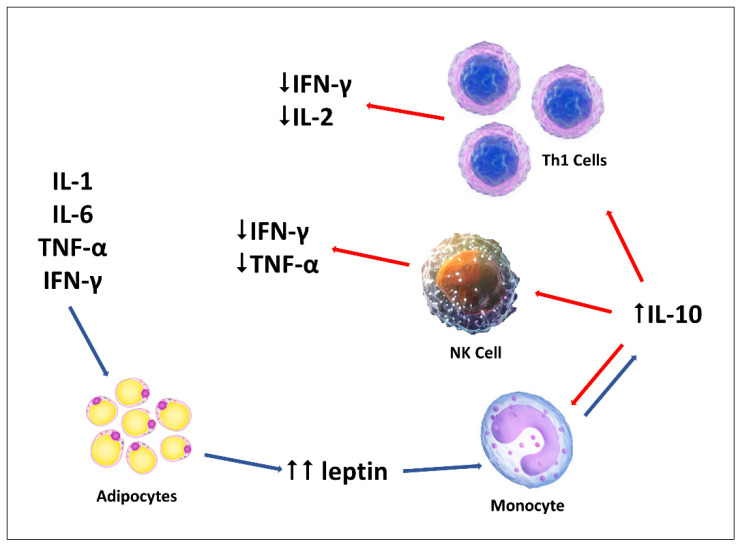
Proposed mechanism of the anti-inflammatory role of leptin in childhood ITP. The red and blue arrows denote down- and upregulation, respectively (adapted from [59]).

Our results demonstrate that leptin plays an active anti-inflammatory role in childhood ITP by increasing the expression of IL-10 by monocytes. We propose the following mechanism: in the acute phase, pro-inflammatory cytokines act on adipocytes, which in turn overproduce leptin. Leptin acts on monocytes, which proliferate and express large amounts of IL-10 among other cytokines, which downregulates the production of pro-inflammatory cytokines by Th1 cells, NK cells and monocytes (Figure 7). Increased IL-10 expression appears to be associated with better prognosis, whereas lower IL-10 levels are associated with disease progression. Large-scale, prospective, long-term studies are needed to confirm the above findings. It could be speculated that children with low IL-10 levels could benefit from leptin-based therapies, which may increase and maintain IL-10 expression and thus prevent disease progression to chronic forms by balancing the immune response. Leptin replacement therapy has been used successfully in humans to treat congenital leptin deficiency and generalized lipodystrophy, and it has also been investigated as a potential treatment for hypothalamic amenorrhea, other forms of lipodystrophy, and diabetes [57,58].

Our results provide evidence for an anti-inflammatory role of leptin in childhood ITP and suggest that elevated levels of a soluble mediator in the acute phase of an autoimmune disease should not necessarily be considered as causative of disease pathogenesis but should instead be investigated for an immunomodulatory function. This new insight into the immunomodulatory function of leptin could lead to future research and revolutionize drug discovery and development for patients with autoimmune diseases.

Our study has some limitations. Although there were no additional procedures or obligations for the study participants, it was still challenging to recruit participants, which is reflected in the relatively small sample size. In addition, the children with ITP in our study were not followed up long-term to assess which entered stable remission and which, if any, eventually relapsed into chronic disease.

In conclusion, the present study demonstrates that leptin is indeed involved in the pathogenesis of childhood ITP, but as an active anti-inflammatory agent by increasing the expression of IL-10 by monocytes. Additional studies are warranted to further investigate the immunomodulatory function of leptin, which may be a target for the treatment of autoimmune diseases.

## 4. Materials and Methods

### 4.1. Study Subjects

Thirty-nine children with ITP were studied. The patients presented to the Department of Pediatrics, Patras University Hospital, and did not suffer from comorbidities. A control group of 33 age, sex, and BMI-matched healthy children was included in the study. Peripheral blood samples were collected from patients before treatment, 24 h after administration of intravenous immunoglobulin (IVIg) (Sandoglobulin, Novartis International AG, Basel, Switzerland) and/or methylprednisolone (Medrol, Pharmacia and Upjohn, Uppsala, Sweden), and at follow-up when remission was achieved. Blood samples from controls were collected once during a routine visit to Patras University Hospital.

### 4.2. Processing of Blood Samples

Peripheral blood samples (0.5–7 mL) were collected in EDTA-coated tubes. The concentration of leukocytes, their populations, and platelets was determined using a hematology analyzer (Cell-Dyn 3700, Abbot Pharmaceutical Co. Ltd., Lake Bluff, IL, USA). PBMCs were isolated from whole blood by centrifugation over a Ficoll-Paque gradient (Biochrom Ltd., Cambridge, UK) and counted in a hematology analyzer (Cell-Dyn 3700). Plasma was collected and stored at −80°C.

### 4.3. Determination of Leptin and TGF-β1 Levels

Leptin and TGF-β1 levels in plasma of patients and controls were measured by ELISA using the Human Leptin Quantikine ELISA kit and the Human/Mouse/Rat/Porcine/Canine TGF-β1 ELISA kit (R&D Systems Europe, Ltd., Abingdon, UK).

### 4.4. Cells, Cultures, and Phenotyping

**Figure 8:**
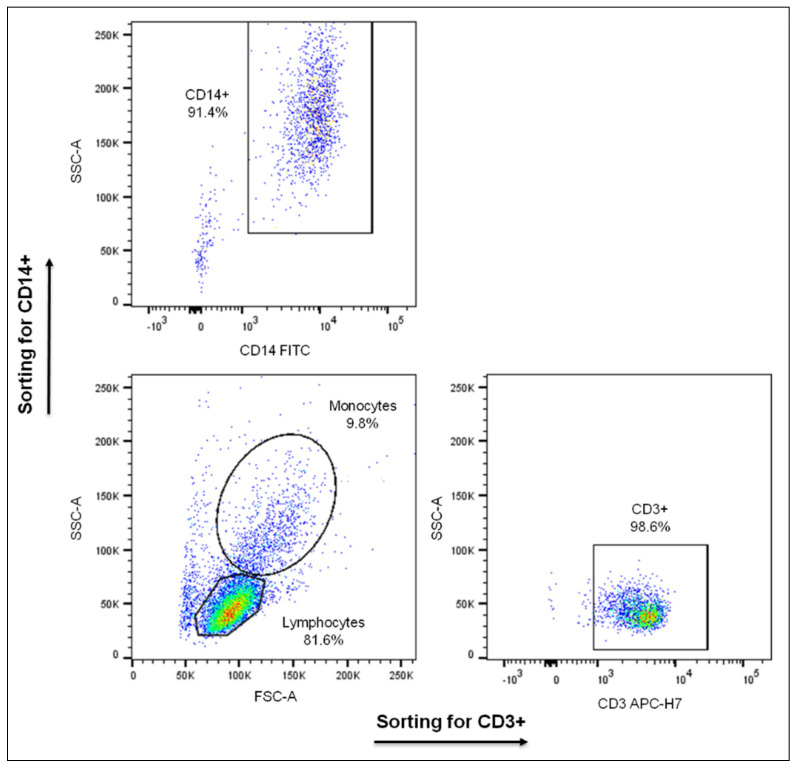
Sorting strategy for the isolation of CD3+ T cells and CD14+ monocytes from PBMCs cultured as indicated in the text. Characteristic dot plots showing the purity, after cell sorting with FACSAria.

PBMCs were washed 3× with RPMI1640 culture medium (Gibco, Thermo Fisher Scientific Inc., Waltham, MA, USA) and resuspended in complete culture medium (CM: RPMI1640 supplemented with 10% fetal bovine serum, 100 U/mL penicillin and 50 µM 2-mercaptoethanol), at a concentration of 10^6^ cells/mL. PBMCs were cultured in triplicate in a 37 °C humidified chamber with 5% CO_2_ for 12 h, in the presence or absence of mitogens (PI: 5 ng/mL phorbol myristate acetate and 1 μM ionomycin) (Sigma-Aldrich, Merck KGaA) or recombinant human leptin (R&D Systems) at a concentration of 200 or 500 or 800 ng/mL. At the end of culture, CD3+ T cells and CD14+ monocytes were isolated by cell sorting using a BD FACS Aria II flow cytometer (BD Biosciences, San Jose, CA, USA). The sorting strategy is shown in Figure 8. The enriched cell populations were used when purity was >95% for T cells and >90% for monocytes. The antibodies used for cell sorting and phenotyping were mouse monoclonal anti-human antibodies CD3-APC-H7 (clone SK7) and CD14-FITC (clone M5E2) (BD Biosciences). Fluorescence-minus-one (FMO) controls were used to identify any background spread of fluorochromes and to establish gating limits. Data were analyzed using BD FACS DIVA v.9 software (BD Biosciences).

### 4.5. Cytokine Gene Expression

The expression of cytokine genes IL-2, IFN-γ, IL-4 and IL-10 was examined in PBMCs and isolated T cells and monocytes by quantitative real time PCR (qPCR). Total RNA was extracted from cells using TRIzol^®^ reagent (Invitrogen; Thermo Fisher Scientific), according to the manufacturer’s protocol. Total RNA was then reverse transcribed into cDNA using the M-MLV Reverse Transcriptase kit (Promega Corporation, Madison, WI, USA). The reaction mixture was incubated at 37 °C for 15 min RT, then at 85 °C for 5 s to inactivate the reverse transcriptase. The qPCR was performed on the Mx3000P QPCR system (Agilent Technologies Inc., Santa Clara, CA, USA) using the 2X SYBR^®^ Green qPCR Master mix (Thermo Fisher Scientific). The following thermocycling conditions were used: initial denaturation at 95 °C for 30 s, 30 cycles of 95 °C for 15 s, 55 °C for 30 s, and 72 °C for 2 min; a final extension at 72 °C for 5 min. Primer sequences used are listed in Table 2. Relative gene expression was quantified by the 2^–ΔΔCq^ method using Mx3000P QPCR software (Agilent Technologies). β2-Microglobulin (β2m) served as an internal reference gene.

### 4.6. Statistical Analysis

Data are presented as means (SD) from three independent experiments. Differences between two groups were evaluated using paired or unpaired Student’s t-test, while differences between multiple groups were evaluated using the one-way ANOVA test followed by Tukey’s post hoc test. Pearson’s correlation coefficient was used to analyze the association between leptin levels and platelet counts. *p* < 0.05 was considered to indicate a statistically significant difference. Data analysis and graphical representation were done using GraphPad Prism 6.0 (GraphPad Software).

## Figures and Tables

**Table 1:** Data of the study participants.

	ITP Patients	Controls
n	39	33
Age (years)	9.29 (1–14)	9.06 (4–14)
Male/Female	20/19	15/18
BMI	18.16 (4.11)	20.78 (2)
Platelet count (×10^9^/L)	27.135 (3–95)	241 (142–349)
Treated with: IVIg	59%	n/a
Methylprednisolone	6%	n/a

n, sample size; age in years, shown as mean (range); BMI, body mass index (kg/m^2^ where kg is a person’s weight in kilograms and m^2^ is their height in meters squared), shown as mean (SD); platelet count, shown as mean (range); IVIg, 2 g/kg body weight (daily dose); methylprednisolone, 4 mg/kg/day, up to 60 mg/day; n/a, not applicable. Data are from patients at presentation.

**Table 2:** PCR primer pairs used for the determination of cytokine gene expression.

Gene	Set of Primers (5′ →3′)	Product Length (bp)
IL-2	CTCACCAGGATGCTCACATTTA	97
	CCTCCAGAGGTTTGAGTTCTTC	
IFN-γ	TGGCTTTTCAGCTCTGCATC	117
	CCGCTACATCTGAATGACCTG	
IL-4	ACTTTGAACAGCCTCACAGAG	74
	TTGGAGGCAGCAAAGATGTC	
IL-10	GCTGGAGGACTTTAAGGGTTACCT	109
	CTTGATGTCTGGGTCTTGGTTCT	
β2m	TCGCGCTACTCTCTCTTTCT	83
	TTTCCATTCTCTGCTGGATGAC	

## Data Availability

The data presented in this study are available on request from the corresponding author.
